# Incongruence between Physician, Patient, and Medical Chart Report of Skin Cancer Prevention Counseling

**DOI:** 10.3390/ijerph19116853

**Published:** 2022-06-03

**Authors:** Natalie H. Matthews, Augustine W. Kang, Martin A. Weinstock, Patricia Markham Risica

**Affiliations:** 1 Department of Dermatology, University of Michigan School of Medicine, University of Michigan, Ann Arbor, MI 48109, USA; 2 Department of Behavioral and Social Sciences, Brown University School of Public Health, Providence, RI 02903, USA; augustine_kang@brown.edu (A.W.K.); patricia_risica@brown.edu (P.M.R.); 3 Stanford University School of Medicine, Stanford, CA 94305, USA; 4 Department of Dermatology, The Warren Alpert Medical School, Brown University, Providence, RI 02903, USA; martin_weinstock_md@brown.edu; 5 Department of Dermatology, Veterans Medical Center, Providence, RI 02903, USA; 6 Center for Health Promotion and Health Equity, Brown University School of Public Health, Providence, RI 02903, USA

**Keywords:** skin cancer, primary prevention, counseling, melanoma, keratinocyte carcinoma, nonmelanoma skin cancer

## Abstract

Skin cancer incidence in the United States has risen rapidly in recent decades, underscoring the need for accessible and effective prevention practices. Skin cancer prevention counseling can lead to increased sun protective behavior and early detection; however, little is understood regarding the frequency and content of counseling among primary care providers (PCPs). We performed multi-center cross-sectional surveys among 53 providers and 3343 of their patients and chart review asking whether skin cancer prevention counseling occurred and details of that counseling. Only 10–25% of patients reported that counseling occurred. Among the providers who reported counseling, there were higher odds that their patients recollected they were advised to use sunscreen or protective clothing, on how to use sunscreen, on signs of skin cancer, to perform a self-skin exam (all *p* < 0.001), and were provided with written materials (*p* < 0.01). Eight percent of prevention counseling was chart documented despite being highly associated with patient and physician recollection of counseling (*p* < 0.001). These results highlight the need for consistent and clear delivery of skin cancer primary prevention.

## 1. Introduction

Melanoma is the deadliest skin cancer [1], and keratinocyte carcinomas (otherwise known as nonmelanoma skin cancer), which include cutaneous basal cell carcinoma and squamous cell carcinoma, are the most commonly diagnosed cancers in the United States [2]. The incidence of melanoma and keratinocyte carcinoma in the U.S. are rising and carry substantial morbidity and mortality as well as significant social and economic burden [1,2,3,4]. Exposure to ultraviolet radiation from the sun is the primary known risk factor for development of skin cancer (melanoma, basal, and squamous cell carcinomas) and precancerous lesions such as actinic keratosis [2,5]. Early detection of skin cancer can improve morbidity and mortality [6]. Skin cancer prevention counseling can lead to increased sun-protective behavior and self-skin examination, which, when implemented, can reduce risk of skin cancer [1,7,8]. Additionally, prevention counseling is a quick, simple, cost-effective method to reduce risk. To effectively confront the growing incidence and healthcare burden of skin cancer, primary care providers (PCPs) must join dermatologists in skin cancer prevention practices. However, dermatologic education and training among medical students and nondermatological residents is variable and does not always include education on primary prevention. To our knowledge, there are no studies that investigate frequency and content of counseling by PCPs, patient, and chart report.

We aimed to investigate the content of skin cancer prevention counseling in the primary care setting, namely details of sun-protective behavior and self-examination, and how often counseling is performed. We also examined what was perceived by the patient versus reported by the physician and what was ultimately charted. We hypothesized that there would be lower patient report of prevention counseling compared to physician and patient record. We also hypothesized that there would be variability in what physicians counseled on.

## 2. Materials and Methods

We conducted telephone, written interviews, and chart review at outpatient general practice clinics across the United States in the Mid-Atlantic, Ohio, Kansas, and Southern California. Baseline data was obtained from a randomized controlled trial of a web-based skin cancer early detection continuing education course (The Basic Skin Cancer Triage (BSCT) curriculum), which has been described elsewhere [9]. This study was approved by the Institutional Review Boards of the Providence Veterans Affairs Medical Center, Rhode Island Hospital, Brown University, and those of all recruitment sites.

We interviewed 53 primary care providers, all of whom had been in practice for more than a year and included general internists, family physicians, or medicine-pediatrics practitioners. We did not recruit from subspecialty clinics, for example, sports medicine or women’s care. We interviewed 3343 patients who were all recruited from participating physician practices and were determined to have minimal risk of melanoma (e.g., no signs of severe photodamage to the skin by providers). Patients were interviewed over telephone within 1–2 weeks of their office visit.

### 2.1. Measures

#### 2.1.1. Demographics

Physicians were asked to provide demographic details including age, sex, ethnicity, degree (MD verses DO), years in practice, training, and previous dermatology training. Patients were asked to provide demographic information including age, sex, ethnicity, education, and household income.

#### 2.1.2. Physician-Reported Counseling Variables

Physicians were asked to characterize their clinical practice, including the following questions regarding performance of skin cancer prevention counseling: “*Please think of a typical month and using the scale below, indicate how frequently you provide patients with (1) counseling and/or (2) resources or materials to assist them to reduce their risk of skin cancer.*” Response options ranged from 1 = never, 2 = sometimes, 3 = about half, 4 = often, and to 5 = almost always.

#### 2.1.3. Patient-Reported Exam Variables

Patients were asked about the content of skin cancer prevention counseling and whether they received it: “During your last visit, as far as you could tell, did your physician: (1) Advise you to use sunscreen? (2) Advise you to protect your skin with clothing or hats? (3) Talk to you about how to best use sunscreen? (4) Help you by explaining the warning signs for skin cancer? (5) Help you by explaining how to do a thorough skin self-examination? (6) Help you by providing written materials about skin cancer prevention?” Response options were binary, 0 = no, 1 = yes. Participants who indicated do not know/refused were coded as missing.

#### 2.1.4. Patient Chart-Extracted Data

Research assistants used a structured abstract form to extract whether skin cancer prevention counseling occurred from the patient’s chart information from the same encounter detailed by patients: 0 = absence and 1 = presence in the chart.

### 2.2. Statistical Analysis

Descriptive statistics included means and standard deviations (SD) for continuous variables and frequencies and percentages for categorical variables. Logit analysis/logistic regression was used to predict the odds of patient-reported outcomes that were binary, with physician-reported variables as the predictor. Analysis was conducted using SPSS version 24 [10]. Significance criterion was set at α < 0.05.

## 3. Results

Demographic descriptive statistics are detailed in Table 1. Among PCPs (*n* = 53), the mean (SD) age was 50.3 (9.9) years and 59.8 (16.9) years for patients (*n* = 3343; Table 1). Providers predominately were allopathically trained (91% MD, 9% DO) and practiced on average for 21.8 (10.6) years; 17% were trained in internal medicine, 4% internal medicine and pediatrics, and 79% family medicine; and 79% of PCPs had previous exposure to dermatology. The patient sample was 58% female, predominately White (84%), non-Hispanic/Latinx (95%), and had completed high school (94%) and earned >USD 40,000 (66%; Table 1).

We found that PCPs conducted skin cancer prevention counseling with variable frequency (Table 2). However, patients of PCPs who reported counseling were more likely to report comprehensive counseling compared to patients of PCPs who did not report counseling (Table 2). However, patient report of counseling was quite low even among those of counseling-reporting PCPs. Only 10–25% of patients reported that any counseling occurred (Table 2). Variability in the content of prevention counseling and discrepancies between patient, PCP, and chart reported counseling was found. Among PCPs who reported conducting skin cancer prevention counseling, <20% of patients reported that they were advised to use sunscreen or protective clothing and <10% on how to use sunscreen, signs of skin cancer, performing a self-skin exam, or were provided with written materials. An increase in PCP-reported skin cancer prevention counseling was associated with (1) a 25.2% increased odds of patients reporting that they had been advised to use sunscreen (95% CI, 1.16, 1.35, *p* < 0.001), (2) 21.9% increased odds of patients reporting that they had been advised on how best to use sunscreen (95% CI, 1.09, 1.36, *p* < 0.001), (3) 23.8% increased odds of patients reporting that they had been advised to wear hats/protective clothing (95% CI, 1.14, 1.34, *p* < 0.001), (4) 37.1% increased odds of patients reporting that they had been explained the warning signs of skin cancer, (95% CI, 1.23, 1.53, *p* < 0.001), (5) 40.4% increased odds of patients reporting that they had been explained how to perform self-skin examinations (95% CI, 1.26, 1.57, *p* < 0.001), and (6) 22.0% increased odds of patients reporting that they had been provided with counseling materials (95% CI, 1.07, 1.39, *p* < 0.001).

In addition, an increase in a score on physician-reported provision of counseling, resources, or materials or reduce skin cancer risk was associated with: (1) a 13.1% increased odds of patients reporting that they have been advised to use sunscreen (95% CI, 1.04, 1.23, *p* = 0.003), (2) 10.0% increased odds of patients reporting that they have been advised to wear hats/protective clothing (95% CI, 1.01, 1.20, *p* = 0.025), (3) 22.9% increased odds of patients reporting that they have been explained how to perform self-skin examination (95% CI, 1.10, 1.38, *p* < 0.001), and (4) 16.1% increased odds of patients reporting that they have been provided with counseling materials (95% CI, 1.02, 1.33, *p* = 0.029).

Medical chart documentation of counseling was exceedingly low. Only 8% of prevention counseling was chart documented despite being associated with patient (Table 2) and physician (95% CI 1.09, 1.31, *p* < 0.001) recollection of counseling having occurred.

## 4. Discussion

Sun protection and self-skin examination counseling is a safe, cost-effective method to decrease risk of skin cancers, including melanoma and basal and squamous cell carcinomas [3,4,5,6,7,8]. Despite its relative ease, we found that one-quarter or less of PCPs we surveyed performed skin cancer prevention counseling. However, among those who did, patients reported that providers often discussed both sunscreen and sun protective clothing use as well as details on self-skin examination. While there was less variability in counseling content than we predicted, there was also less reported and chart documentation of counseling than we anticipated.

This low patient report of skin cancer prevention counseling may in part be explained by potential limitations in comprehension; however, the detail reported by patients who did report counseling would suggest otherwise. Differences in counseling style and provider comfort with conducting skin cancer primary prevention may have also contributed to lower report of prevention counseling. Dermatology curriculum is not standardized or even included in all medical student and resident education. In our sample of 53 physicians, 79% had reported receiving some degree of dermatology education; however, we did not assess the extent to which they had been trained in skin cancer prevention counseling. In a survey of 659 U.S. medical students, >2/3 of students had no clinical exposure to dermatology [11]. In survey among 342 internal medicine, family medicine, pediatrics, and obstetrics/gynecology residents across seven medical schools and four residency programs in the U.S., >3/4 of residents had never been trained in skin cancer prevention counseling [12]. Our findings reflect insufficient and inconsistent training in skin cancer counseling practices and the need for its mandatory education to ensure quality primary prevention in the primary care setting.

Compared to PCP self-reported performance of counseling, considerably fewer PCPs marked the patient chart as having completed prevention counseling. However, among patients whose charts indicated that counseling had been conducted, they reported higher odds of all counseling topics having been discussed, including provision of written materials. The relatively low chart documentation of counseling highlights the need for more rigorous enforcement of accurate charting.

To our knowledge, this is the first study examining PCP, patient, and chart report of skin cancer prevention counseling. Our exploratory cross-sectional survey highlights potential room for improvement among general practitioners to regularly integrate skin cancer prevention counseling into their care routine. While potential impediments include provider education and training, even referral to web and text-based materials through trusted sources such as the American Academy of Dermatology may be sufficient to promote sun-protective behaviors and possibly improve self-detection as well.

Reliable and consistent skin cancer prevention counseling from PCPs may have a profound impact on skin cancer incidence in the U.S. Ultimately, there may need to be national efforts to promote awareness of sun-protective behaviors and warning signs for skin cancer. International examples of successful primary prevention campaigns against skin cancer include the Sunsmart campaign. Sunsmart, launched by the Australian government, emphasizes sun protection and warning signs of skin cancer from primary schools to the workplace and has played a notable role in decreasing Australia’s national skin cancer incidence [13,14]. Australia’s targeted methods of primary prevention [13,14] provide an example that the U.S. could potentially model.

Our study was subject to limitations in data collection, including variable duration of time lag between patient visit and surveys, and a modest provider sample size. Furthermore, detail of clinic work was not provided beyond selection of outpatient general primary care practices, which may contribute to variability in counseling frequency. Conversely, strengths of our study included recruitment of participants from geographically diverse centers and independent assessment of provider reported practices, objective assessment of charting, and patient report of their encounter, which was critical to assess independent contributions of each data source.

## 5. Conclusions

Our study highlights how little counseling occurs in the primary care setting as well as the variability in content despite the need for increased primary skin cancer prevention [1,2,7]. Our results may reflect differences in patients’ ability to absorb content during clinical evaluation and interpretation of prevention counseling. To improve skin cancer prevention and measure impact, investigators must be aware of considerable differences among how data are ascertained. Despite its limitations, our small but novel cross-sectional survey underscores the need for consistent, routine prevention counseling and clear clinical expectations regarding skin cancer education from PCPs. Future directions for study include examining frequency and content of counseling on a larger population scale and how sex, race, age, level of education, and training may impact skin prevention counseling between PCP and patient report.

## Figures and Tables

**Table 1:** Demographic characteristics of physicians and patients.

	Physicians (*n* = 53)		Patients (*n* = 3343)
Mean Age	50.26 ± 9.94		59.8 ± 16.9
Sex			
Male	34 (64.2)		1418 (42.4)
Female	19 (35.8)		1923 (57.6)
Race			
Asian	8 (15.1)		104 (3.2)
African American	0 (0)		294 (9.1)
White	44 (83.0)		2726 (84.1)
Other *	1 (1.9)		117 (3.6)
Hispanic/Latinx			
Yes	1 (1.9)		178 (5.3)
No	52 (98.1)		3157 (94.7)
Mean years in practice	21.8 ± 10.55 (range: 3–49 y)	Education	
		<High school	208 (6.2)
Training		High school/GED	822 (24.6)
Internal medicine	9 (17.0)	Some college	847 (25.4)
Internal medicine and pediatrics	2 (3.8)	College	902 (27.0)
Family medicine	42 (79.2)	Postgraduate	554 (16.7)
Previous dermatology training		Income	
Yes	42 (79.2)	<USD 20 k	431 (13.8)
No	8 (15.1)	USD 20–40 k	647 (20.7)
Did not answer	3 (5.7)	USD 40–80 k	952 (30.5)
		>USD 80 k	996 (31.9)
		Did not answer	96 (3.1)

Physicians are primary care providers. Data expressed as *n* (%) or M ± SD. Complete case analysis was conducted. * patients who self-identify as a race other than the above listed demographic categories.

**Table 2:** Logit analysis comparing patient-reported, primary care physician-reported, and patient chart documentation of skin cancer prevention counseling.

	Physician-Reported Advice for Skin Cancer Risk Reduction			Physician-Reported Provision of Prevention Resources or Materials			Patient Chart Indicated Counseling		
Patient-reported counseling		Score (M ± SD)	Odds Ratio, 95% CI		Score (M ± SD)	Odds Ratio, 95% CI	No	Yes	Odds Ratio, 95% CI
Advised to use sunscreen	No (*n* = 2617)	2.91 ± 1.09	1.25 (1.16, 1.35) ***	No (*n* = 2617)	2.18 ± 1.03	1.13 (1.04, 1.23) **	2407 (82.7)	150 (60.7)	3.09 (2.36, 4.07) ***
	Yes (*n* = 611)	3.19 ± 1.08		Yes (*n* = 611)	2.30 ± 1.01		503 (17.3)	97 (39.3)	
Advised how best to use sunscreen	No (*n* = 2971)	2.95 ± 1.09	1.22 (1.09, 1.36) ***	No (*n* = 2971)	2.18 ± 1.03	1.06 (0.94, 1.19)	2710 (92.8)	201 (80.7)	3.08 (2.18, 4.35) ***
	Yes (*n* = 268)	3.19 ± 1.08		Yes (*n* = 268)	2.25 ± 0.97		210 (7.2)	48 (19.3)	
Advised to wear hats/protective clothing	No (*n* = 2628)	2.92 ± 1.09	1.24 (1.14, 1.34) ***	No (*n* = 2628)	2.17 ± 1.03	1.10 (1.01, 1.20) *	2402 (82.5)	165 (66.3)	2.41 (1.82, 3.18) ***
	Yes (*n* = 602)	3.18 ± 1.10		Yes (*n* = 602)	2.27 ± 1.02		508 (17.5)	84 (33.7)	
Explained warning signs of skin cancer	No (*n* = 2951)	2.93 ± 1.09	1.37 (1.23, 1.53) ***	No (*n* = 2951)	2.18 ± 1.03	1.09 (0.97, 1.22)	2691 (92.2)	195 (79.3)	3.09 (2.21, 4.32) ***
	Yes (*n* = 285)	3.32 ± 1.13		Yes (*n* = 285)	2.28 ± 0.99		228 (7.8)	51 (20.7)	
Explained how to do self-skin exam	No (*n* = 3052)	2.93 ± 1.08	1.40 (1.26, 1.57) ***	No (*n* = 3052)	2.17 ± 1.03	1.23 (1.10, 1.38) ***	2695 (92.5)	208 (83.5)	2.43 (1.69, 3.50) ***

* *p* < 0.05; ** *p* < 0.01; *** *p* < 0.001.

## Data Availability

Data available on request due to restrictions, e.g., privacy or ethical.
